# Key to Cholera

**Published:** 1848-09

**Authors:** G. W. Maxwell

**Affiliations:** Calcutta


*Key to Cholera.* By G. W. Maxwell, M. D. Calcutta.

PREFACE.

This "Key to Cholera" is a condensed epitome of the practically useful part of a work on the same subject now passing through the Calcutta press; but the uncertainty of its appearance, at a time when cholera is raging here and elsewhere, has induced me to publish this key without further delay.

In this paper I have omitted all that might not prove directly and practically useful, and I have confined myself to giving a natural sketch of the disease, and pointing out the natural indications of cure. I am enabled to do this principally from the circumstance of having had three attacks myself at different intervals of time; and it was only after and from the last (the severest) that I derived any decided knowledge regarding the real nature of the disease.

My views, I was happy to find, corresponded with those of Sydenham, whose principles of treatment had been at last adopted in England. Among other important symptoms, however, the spasms, I find, have not been satisfactorily explained in any English author that I have met with; their nature was, however, revealed to me during my last attack, and their explanation will be found in the other treatise. Suffice it, for all practical purposes, here to say, that irritants to the extremities for the relief of spasms are unnecessary and injurious, and that their cure, equally with that of all the other symptoms in the chain, depends upon the natural system of treatment. The natural system I call that which is indicated and at once explained by the symptoms; that which is the spontaneous, instinctive, and successful choice in the moment of pressing urgency and danger; that which, in spite of all laboured theory or hypothesis, remonstrance or ban, is chosen and eagerly demanded by the sufferer; which in theory is denounced as death, but which in practice enables the patient to rise up with renewed energy and life. Such was the natural system I adopted in my own case, solely directed thereto by the natural instincts, in opposition to all that I had learned from books, or fabricated in my brain from theory or hypothesis. I found all wanting at the time I most required their assistance. But Nature, the best and kindest physician, directed me to the natural system of cure, whereby I was not only quickly cured, but meanwhile escaped the torments of that destroying thirst which is present in every case.

Now, the chief object in an epitome like the present is to endeavour to explain the principles on which the cure depends, rather than waste time in the enumeration of various remedies which would serve but to perplex and annoy the judgment. With this object in view, therefore, I will briefly describe the progress of disease, and the corresponding symptoms, from which, as a matter of course, the principles of cure will be made apparent; but I will also assist the conclusions of the reader by a reference to my own case.

### THE NATURE OF CHOLERA.

*The Progress of Symptoms.*—What is cholera? is a question that has been asked a million times.

Cholera is the first stage of fever; the fever of a particular locality—the endemic fever, or the epidemic fever.

Fever is made up of various stages; the collapse stage, the shivering stage, the hot stage, and the sweating stage. All or each of these may be morbidly increased, constituting apparently different diseases, but in reality linked together in inseparable union. It is the morbid increase of the first of these that I have now briefly to consider, viz., cholera morbus.

Here the fever never rises higher, it never reaches the shivering or the hot stages; if it does, it is no longer cholera; the fever has passed from the collapse into the other stages. Those who have had ague will comprehend the term "collapse of fever." They will recollect having had the paleness of the hands, feet, and countenance (and these generally tipped with blue;) they will recollect the cold smooth feeling of the hands, the nervous sensation about the chest and stomach, and extending over the system (together with others mentioned in the treatise.) These, all or partly present, constitute what I call a "collapse of fever;" and this collapse of fever (in excess) is cholera morbus.

During the prevalence of the epidemic constitution, if an individual sojourn in a locality notoriously febrile, he will imbibe (what I will call for the easier comprehension of the reader) the epidemic leaven or ferment. Now, this ferment will take some time to display its full action, varying according to the quantity taken into the system; but it is generally in the middle of the night following that the effects are displayed; and it is an equal chance whether the individual sinks into the first or collapse stage, or rises from it into fever; hence the explanation of those cases, found in the morning in a high state of fever, which had been first reported as instances of cholera. The development of the stages of fever entirely depends on the changes the leaven has effected. If this change has been such that the blood has become too thick to flow through the lungs, then, as a matter of course, the collapse stage is developed in excess; in other words, cholera asphyxia is exhibited. The blood, unable to pass through the middle passage into the arteries, collects and swells out the veins, giving that deadly or blue colour to the skin. When the vomiting and spasms come on, this mass of blood in the veins is squeezed with great force, and hence the clammy moisture that is forced from every part during these fits. There is no pulse, because there is no blood in the arteries. There are also lethargy and languor, and oppression in breathing, caused by the blood being all collected in the veins. These make up the principal links of the chain of mechanical symptoms. The other train of symptoms and associate symptoms arises directly from the stomach and bowels. I cannot say which are the most important; the neglect of either may be fatal. They, like the former, spring from the influence of the epidemic leaven. When the blood begins to thicken, that same moment all the functions begin to go wrong. The most important of all the functions, digestion and assimilation, are the first to feel the influence ; in fact, it is difficult to define priority ; the influence must be immediate, being part of the same circle. The derangement of these functions and the depravation of the blood advance mutually, as a matter of course ; neither the one furnishing secretions to the bowels, nor the other nutrition to the blood. The inevitable, invariable consequence of this is the establishment of fermentation of the contents of the stomach and bowels ; the abdomen becomes swelled, and the stomach and bowels more or less uneasy ; and this uneasiness increases exactly in proportion to the completeness of the changes the alimentary matters undergo. Nausea advances rapidly, followed by a vomiting and purging ; and, if there is not a free discharge both ways at first, spasms are induced by the irritating fermenting matters remaining in the intestines ; if these are in the stomach or other portions of the bowels, the spasms will be in the chest and upper extremities ; if in the lower part of the canal, the spasms will be in the inferior extremities. Examination after death reveals the origin of these spasms in the mucous membrane of the bowels ; it is found more or less destroyed in various parts, or covered with ulcerations in protracted cases. The contents of the bowels are found in a putrid state ; there are no healthy secretions, and not a particle of bile—the preserving fluid of the intestines, the register of putrefaction. The moment it disappears, fermentation and putrefaction advance rapidly. Its absence is one of the links in the great chain ; as also are all the effects resulting therefrom. There is not a single secretion carried on in fully formed cholera—for this plain reason, that there is no circulation ; the blood is too thick to pass through the middle passage into the arteries ; it remains in the veins, and during each fit of vomiting and spasm it is squeezed, as in a cheese-press, and the clammy moisture forced from it at all parts. Hence observe the chain of actions : the leaven leavening the mass, thickening of the blood, stoppage of digestion, fermentation of the alimentary matters, irritation of the mucuous membrane, vomiting, purging and spasm, all reacting, as it were, on the first symptoms, and increasing the thickening of the blood ; all in fact, parts of the chain linked together in inseparable union.

PRINCIPLES OF CURE

From the foregoing brief illustration of the progress of symptoms, may be readily inferred the principles of cure indicated in the treatment; and the reader has, no doubt, in part formed his own natural conclusions thereon. I will assist him in the correct formation of these by the recital of my own case, from which alone it was that I myself derived any decided views regarding the nature of the disease or the principles of cure.

I must necessarily spin out the case to elucidate the subject, but shall do so as concisely as I can, consistent with the object in view. I had the last attack of the disease about this time last year, in the Northern Circars, where cholera was raging all along the great military roads north and south. My attack was in the evening, between seven and eight (rather unusual with such attacks, which take place generally a little after midnight; the reason of this I have considered in the treatise.)

I had dined rather heartily about three o'clock. After I had gone out, about half-past five, I experienced a disagreeable feeling of distention across the stomach, which, however, nearly gradually wore off for the time, but partly returned, with the addition of nausea, on reaching home. After a while, vomiting suddenly came on, attended with inordinate straining. I drank copiously of cold water to relieve this straining, and after a while it ceased; but it returned again.

I had again recourse to copious draughts of cold water, and after a time obtained relief; but in about a quarter of an hour the nausea, with the vomiting and excessive straining, again returned, and at the same time a sudden irresistible call to stool, when I passed without any exertion a copious, watery, putrid evacuation. I was now fainting, and threw myself down, bathed in cold clammy sweat. A few more involuntary evacuations, and spasms in the toes, feet, and legs (observed first in the right then in the left leg,) convinced me that the disease had no tendency to stop.

I now began to run over in my mind all that I had read in books about the disease, and the best methods of treatment.

*Bleeding* first presented itself; just at the same moment I felt a spasm in the right leg (which was held already by two domestics,) and I immediately began to couple the two together in mind, when, to my astonishment, the spasm changed from the right leg to the left, followed, or rather accompanied by an evacuation, and then a cessation of the spasm. This immediately pointed out to me the nature of the spasms and the cause of the evacuations, viz., that they proceeded from irritation of the mucous membrane, produced by matters resting in the tube; hence I perceived that bleeding could not relieve these particular symptoms; and so I now, in rapid succession, turned my attention to laudanum, but I find the same reasoning apply to it; I knew it would lull for a time, but what security against a recurrence of the vomiting, and purging, and spasm, while semi-stupified by the drug and the disease? What security that it would stop the fermentation of the contents of the bowels, which were irritating the mucous membrane, causing vomiting, purging and spasm, sinking, and loss of voice? None—and if none, thought I to myself, in my case, where the last meal had been nearly all rejected, then what security in those, where the whole of a large meal has passed down into the small intestines?

I next thought of calomel and opium, but here, again, I found the same reasoning apply. It is recommended to be given in both large and small doses: which was I to follow? I knew not, because I saw not the principle of its exhibition in my case. I could not see how calomel and opium resting in the stomach could remove from the colon a mass of prawns, potatoes or plums, that might be resting there, undergoing fermentation and keeping up irritation—the cause of all the consecutive train of symptoms.

My thirst now was becoming overpowering, my tongue and throat were glued, as it were, together, and I looked in vain to bleeding, calomel, and opium for a relief to this most distressing of all symptoms. I had previously partaken freely of cold water, at first to ease the straining, and clear the stomach (a most important point;) but, as the disease began to be developed, I abstained from its use, under the borrowed impression of its being death in this disease. I am ashamed to say that I was ignorant of, or had forgot Sydenham's principles of cure in cholera.

The thirst, however, became worse and worse, and I determined to relieve it at all hazards, and not add misery to death. Having made up my mind, the next point was the choice of the particular beverage; there was plain water, whey and barley-water, gruel, congee, &c., wine and water, brandy and water, &c. To the last of these I had a repugnance, as every one has in fully formed cholera, and the others would require time and direction for their preparation, which my disease was not able to afford or I to give. Whilst thus ruminating my eye accidentally fell upon a packet of effervescing soda powders standing among a crowd of other remedies and nostrums on the table. It immediately took my fancy; it struck me as the very thing I wanted, and without further delay pointed to it, and made signs for a copious draught thereof. It was soon made and soon swallowed; it was extremely refreshing and agreeable, and the thirst was allayed; no nausea succeeded, and the pleasing anticipation remained of having a repetition of the draught whenever I desired. This I was not long in desiring; in fact, almost immediately after I swallowed another, and continued repeating it whenever the thirst became urgent. Instead of retrogading or remaining stationary, I began to improve; the stools became easier, and the spasms less vigorous and vicious.

I experienced an inclination to sleep, a desire to be covered up, and for something hot to drink (these are the best signs, they point out the disease escaping from the collapse stage.) I had a large tumbler full of very warm but weak brandy and water made, and drank it off. I fell asleep and had five or six hours of profound repose. I awoke bathed in perspiration, and with the exception of a little stiffness and considerable thirst, I felt perfectly well. The thirst was again relieved by the effervescing draughts, and I followed up the principle with a couple of dishes of that most delectable and pre-eminent of all stomachics, tea.

Here ends my case with remarks thereon and inferences therefrom. I have only attended to the display of the principle of cure as best I could. Were I to begin with remedies, I might write till this time next year without advantage.

I do not say that the effervescing draught is the only cure, but it is one which carries out the principle as well as any I know, and it is agreeable and refreshing, and allays the thirst; can be taken in any quantity and is efficacious. I have given it in various instances, in every stage, always with advantage to the disease and gratification to the patient; but from these I do not only judge; it is from having taken it myself, as I have described, that I feel authorized thus to speak regarding it.

Bleeding, both general and topical, may be necessary in cholera when there is much oppression, restlessness, pain, spasm, blue skin, or asphyxia; neither were necessary in my case. People do not die from being bled, even when unnecessary, but from bleeding being trusted to alone, while the principle of cure I have pointed out is not followed up.

Sinapisms and blisters to the legs, &c., for the relief of spasms are unnecessary; the origin of the spasms is in the intestines, as I have pointed out. Hot fomentations to the loins and stomach relieve the spasms of the legs. The wishes of the patient should be strictly attended to; Nature is the best physician; if he wishes for cool air he must have it; if he desires to be covered up he must be so; many perish from being too much covered up at first, when the fresh air would revive them (see the treatise for an explanation of this and other circumstances.) Calomel and opium may be necessary in some cases; in mine they were not, therefore were not used. From it may be defined the nature of those cases that might require their adminstration. No harsh remedies will do in cholera; all must be of the mildest description—such as will pass in quantity gently along the bowels to remove the fermenting matters; and, above all, they must be such as will be relished and eagerly desired by the sick, and such as can constantly be taken for the relief of the urgent destroying thirst, constantly present from the commencement of the disease.

### RECAPITULATORY SUMMARY.

I have endeavoured to make this key as concise as possible, consistent with utility. I think it will be found to embrace the most important points connected with the disease. Of all these the irritation of the stomach and bowels claims the first rank; it is caused by the presence of fermented matters. The cure cannot be accomplished till they are removed, or their acrimony blunted; and this must be effected in the gentlest manner by copious diluents, as I have pointed out. I took, in my own case, the effervescing draughts, and

I found them answer admirably; they were delightfully refreshing, and they passed gently downwards, removing the irritation in the bowels. I was solely guided by the thirst; it no sooner returned than I swallowed another tumblerful of the effervescing draught. After taking fifteen at least of these, always with relief and gratification, the disease began to rise through the other stages, indicated by the wish to be covered up, and for something hot to drink, as I have already described.

I will not say a word on the question of bleeding; it is impossible to lay down a fixed rule on this head, or to explain, within the limits of an epitome like this, all the circumstances connected with it; suffice it to say—1. That, if the natural diluent system is early had recourse to, bleeding will seldom be necessary. 2. That bleeding alone will not cure the disease,—for this plain reason, that it cannot remove the fermented irritating matters from the bowels. 3. That the natural diluent system, if early and steadily persevered in, not only removes this irritation, but likewise prevents the further thickening of the blood. If these conditions and their effects, however, from neglect of diluents or other causes, have become urgent, let blood be taken away; it will flow if diluents are now freely given, and the surface kept moist, according to the wishes of the patient.

One parting word, and I have done. Neglect not the desires of the sick. Behold the mortality within these few days. How many in the spring of life have been swept away! What has thewithholding of liquids, covered up, and friction done, save adding misery to death!

Appendix.—Now, in tributary justice to the memory of the illustrious Sydenham, I must add the following quotation from his celebrated works, by which it will be perceived that I have done little else save re-echo the treatment of cholera pursued by him in England nearly two hundred years ago.

*Sydenham's Treatment.*—Let a chicken be boiled in about three gallons of spring-water, so that the liquor may scarce taste of the flesh. Several large draughts of this are to be drunk warm, or, for want of it, of posset drink. At the same time I order a large quantity of the same to be given at several times successively by way of clyster, till the whole be taken in and discharged by vomiting and stool. In this manner the sharp humours are either evacuated, or their acrimony blunted. When this business is over, which requires three or four hours, an opiate completes the cure.

*Swan's Observations*, 1742, *in Sydenham.*—The general indications of cure in this disease are—1st, to correct and soften the acrimonious peccant matter, and fit it for expulsion, and, if there be occasion, to expel it by art; 2d, to check the violent motions in a proper manner; and, 3d, to strengthen the weakened nervous parts.

The discharge of fermented and corrupted diet should be encouraged by gentle emetics, lenient cathartics, and plentiful dilution with whey, thin water-gruel, the small chicken-broth recommended by our author, and the like, and afterwards give strengthening medicines to complete the cure.

Cold water is esteemed an excellent remedy in Asiatic cholera; and is said to be so much the more effectual, the warmer the climate, season, and constitution of the patient be. It mitigates and takes off the violent heat, dilutes and blunts the acrimony of the humours in the bowels, &c.—*London Med. Times.*

